# Supplementary figures and images for: Active foot placement control ensures stable gait: Effect of constraints on foot placement and ankle moments
Source: PLoS One. 2020 Dec 17;15(12):e0242215. doi: 10.1371/journal.pone.0242215 (PMC7746185; doi:10.1371/journal.pone.0242215)

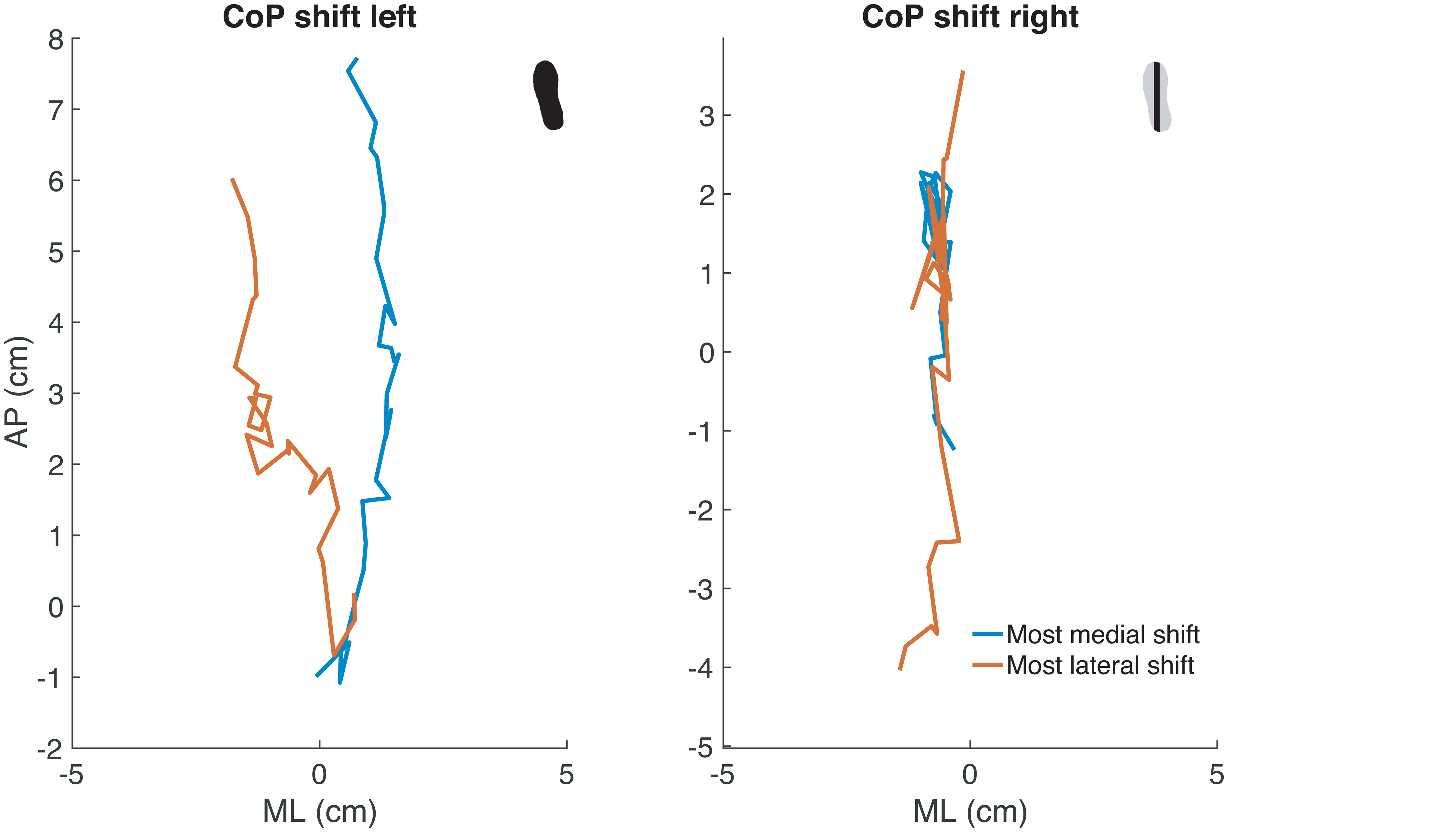

Supplement: S1 Fig — The most medial (blue) and most lateral (red) shifts are plotted, showing divergence of these shifts when unconstrained (left panel) as compared to overlaying shifts when constrained (right panel). The mediolateral CoP shift is limited by a ± 1-centimeter ridge underneath LesSchuh (Fig 2). The figure presents an example of participant 18. Ankle moment constraint–effect of “LesSchuh”. (TIF) [file pone.0242215.s001.tif]

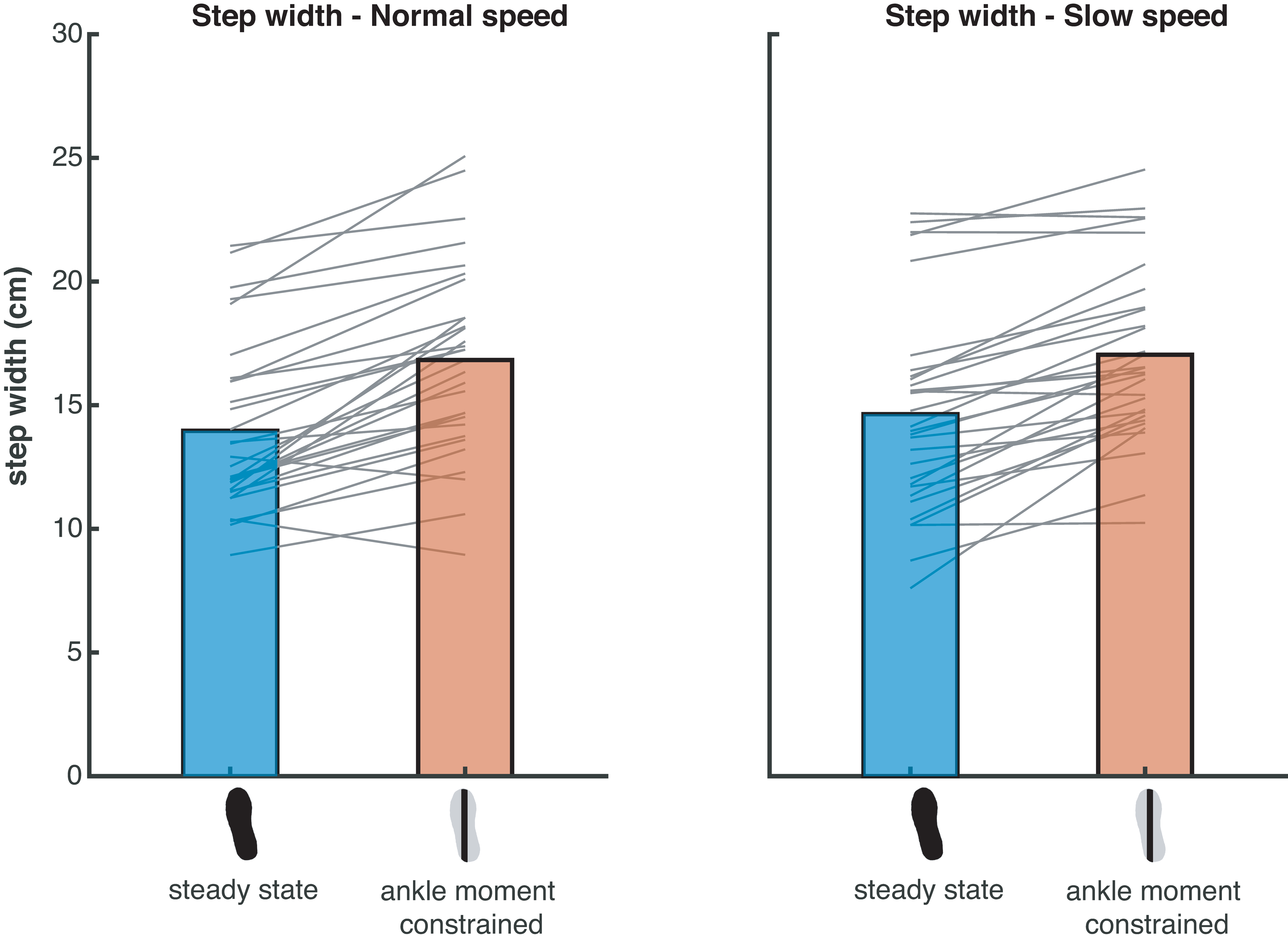

Supplement: S2 Fig — Blue and red bars represent respectively the steady-state walking and ankle moment constrained conditions. The grey lines connect the individual data points. An exploratory Bayesian repeated measures ANOVA, including the steady-state walking and ankle moment constrained condition at both speeds, revealed that the best model included only the factor “Condition” with extreme evidence as compared to the Null model (BF10 = 1.610 * 1015). Post-hoc analysis provided extreme evidence supporting an increase in step width at both speeds to compensate for the ankle moment constraint (BF10 = 1.064*1013). The influence of the ankle moment constrained condition on step width. (TIF) [file pone.0242215.s002.tif]

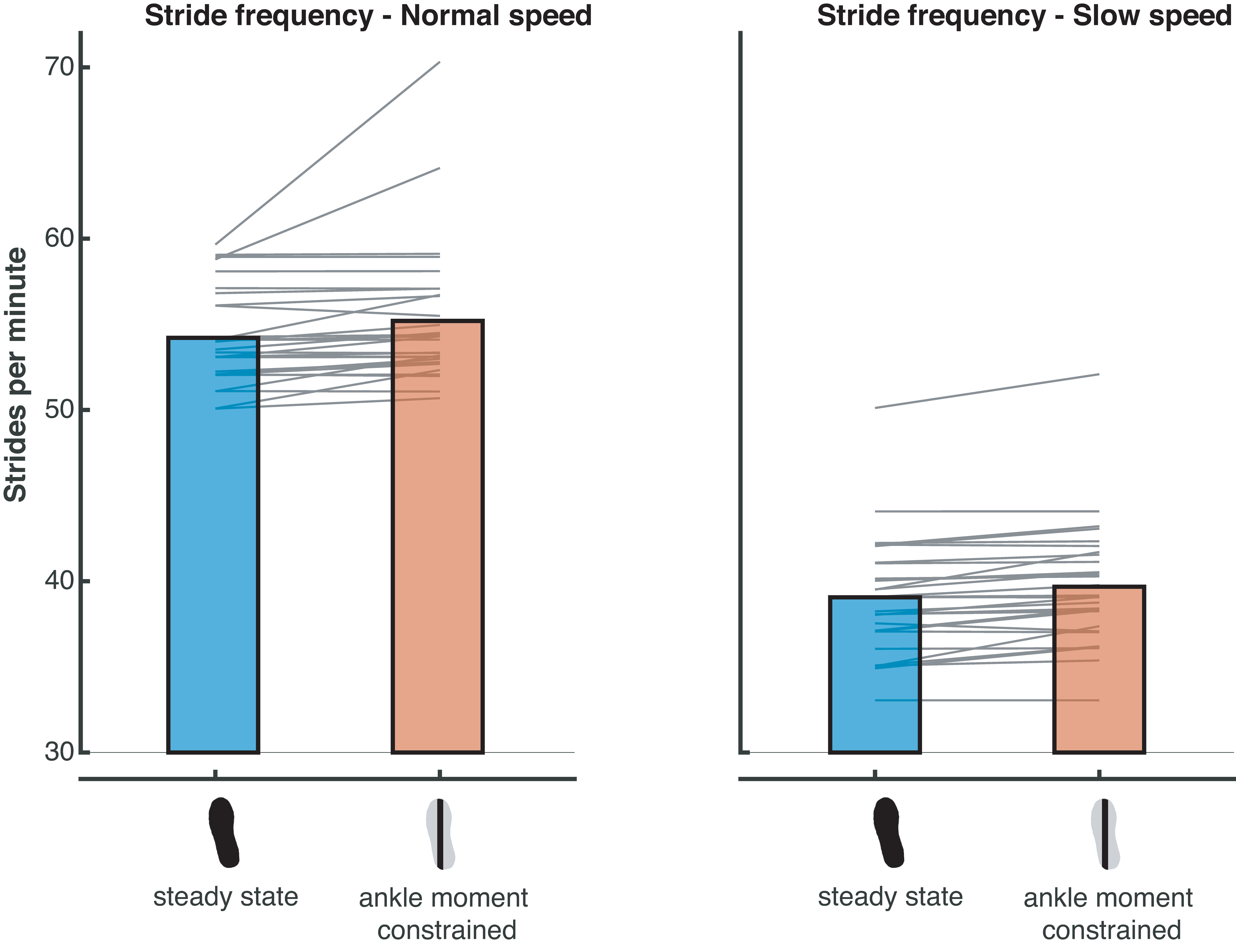

Supplement: S3 Fig — Blue and red bars represent respectively the steady-state walking and ankle moment constrained conditions. The grey lines connect the individual data points. As an exploratory analysis, as well as a protocol check, Bayesian repeated measures ANOVA, including the steady-state walking and ankle moment constrained condition at both speeds, revealed that the best model included the factors “Condition” and “Speed”. Post-hoc analysis provided extreme evidence (BF10 = 7.959*1042) indicating that stride frequency increased in the ankle moment constrained conditions as compared to steady-state walking. The influence of the ankle moment constrained condition on stride frequency. (TIF) [file pone.0242215.s003.tif]

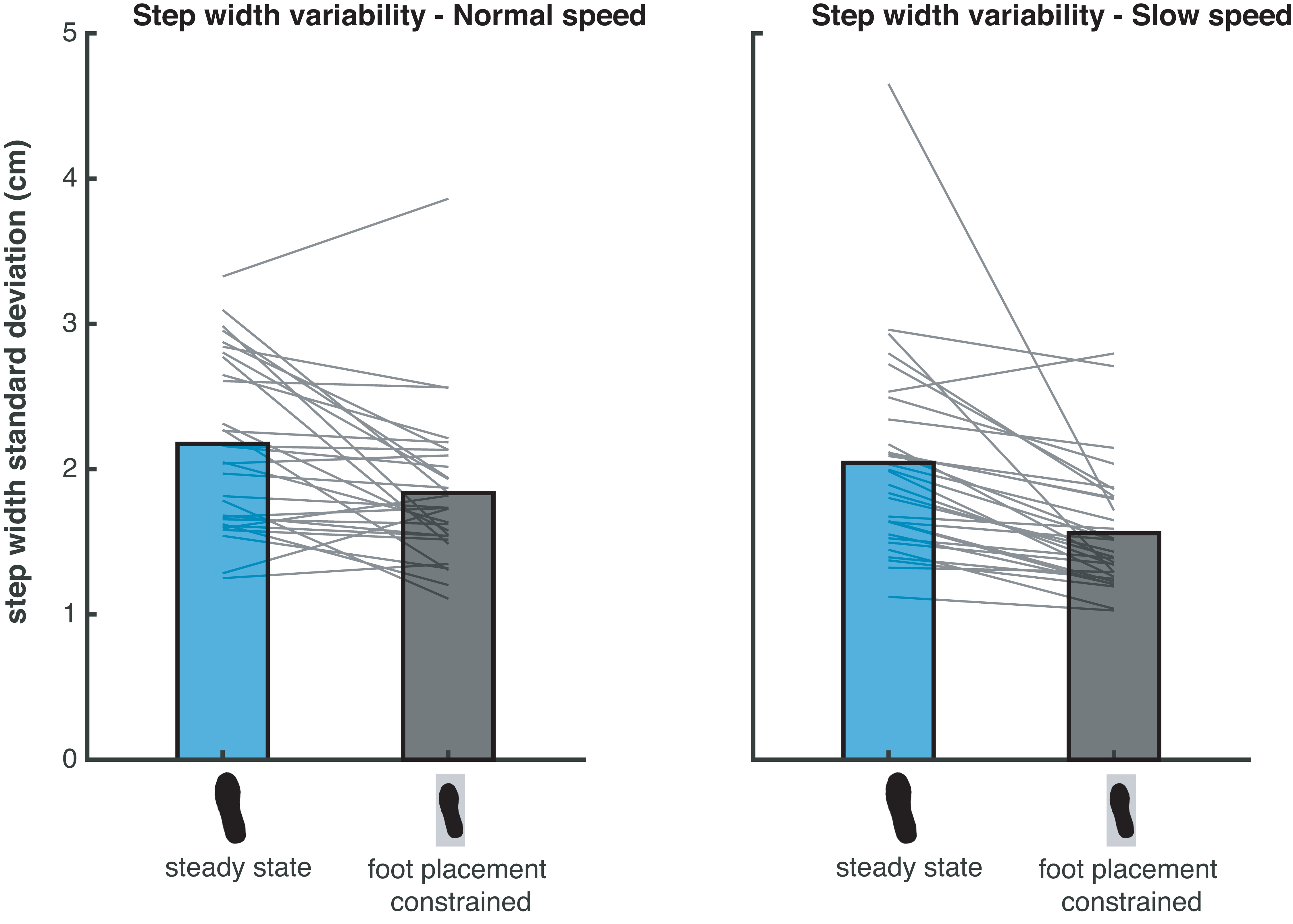

Supplement: S4 Fig — Blue and black bars represent respectively the steady-state walking and foot placement constrained conditions. The grey lines connect the individual data points. Bayesian repeated measures ANOVA, including the steady-state walking and foot placement strategy constrained condition at both speeds, revealed that the best model included the factors “Condition” and “Speed”, with extreme evidence as compared to the Null model (BF10 = 590646.967). Post-hoc analysis provided extreme evidence for a lower step width variability in the foot placement constrained condition as compared to steady-state walking (BF10 = 55714.494). When comparing between speeds, a two-tailed Bayesian paired samples t-test provided extreme evidence demonstrated that in the foot placement constrained condition, the step width variability remained higher at a normal walking speed as compared to the slow walking speed (BF10 = 2091.388). Effectiveness of the foot placement constraint: step width variability. (TIF) [file pone.0242215.s004.tif]
