# Supplementary material for: Active foot placement control ensures stable gait: Effect of constraints on foot placement and ankle moments
Source: PLoS One. 2020 Dec 17;15(12):e0242215. doi: 10.1371/journal.pone.0242215 (PMC7746185; doi:10.1371/journal.pone.0242215)
Supplement: Attachment — Submitted filename: PLOS_Bruijn_Review.pdf [file pone.0242215.s005.pdf]

<u>Review of “Step width and step frequency to modulate: active foot placement control ensures stable gate”</u>

My summary of the paper:

In this paper, the author inquires how active foot placement control is compensated when the ability to modulate ankle moment is constrained or when step width is constrained. The author finds that instead of any modulation in foot placement control, the average step width and step frequency are changed. As the author admits, this does not necessarily mean that active foot placement does not adapt when constrained, because the ankle moment constraint imposed here could have inadvertently constrained the ability for active foot placement as well. The paper also replicates two results from past papers.

A summary of my review:

The paper is well-written and the results are discussed and interpreted with care and insightfully, in the context of the literature. Also, it is to be appreciated that the author appropriately preregistered their methods and declared deviations from preregistrations. It is great that the author will make the data available. One main suggestion I have to the author is to explore if there were any systematic trends in the CoM and foot placement states, which needed to be accounted for when demeaning. Secondly, I suggest the author compare the step width constrained control results to Perry and Srinivasan 2017, which doe a similar thing. In the writing, some further details and justifications need to be provided in the methods section of the paper and I provide specific suggestions for these below.

Detailed review (key changes suggested):

When the author says that the hip states and foot placement variables were “demeaned”, do they account for any systematic trends in the states over time? If not, I suggest the author de-trend these variables and perform the regression models again after de-trending to see if this changes the results. This is essential because, if there is a systematic trend over time in these states, it will affect the central results (the control gains) in this paper. I believe this will not take much time to add as it will just need the addition of one line of code.

The constraint condition in which the step width is constrained is similar to the constraint in Perry and Srinivasan 2017. I think it would be appropriate for the author compare and discuss the results here in comparison the results in that paper.

There are some additional details and justifications needed in the methods section. (i) Why did the author choose to constrain the step frequency? Neither of the papers this paper replicates use a metronome (Wang et al. and Rankin et al.). So, the author should justify this experimental choice to constrain step frequency. Also, it is unclear if the metronome was used for all the conditions i.e. steady-state, ankle constraint, and foot placement constraint. (ii) The author use a certain method to calculate the CoM (using regressions), could they provide more details about how the CoM was calculated or refer to a previous paper that calculates the CoM using a similar method? (iii) Further details need to be provided on the setup used for projecting beams onto the treadmill for the foot placement constraint condition. (iv) I suggest the author add a glossary explaining all the variables used in the paper in a single location.

Line-by-line comments (mostly minor):
<u>Title:</u> Can the title be revised for clarity? “step width and step frequency to modulate” sounds a bit unclear. Also, the focus of the paper is on the added constraints so that should feature in the tile in some way.

Abstract:
In line 20, “ankle strategy” is unclear. Maybe say “ankle moment modulation strategy” to show the link to the previous sentence.

In line 31, I think it is misleading to say “foot placement control was not tightened”, because a change in average step width and step frequency (as was found here) is also achieved through foot placement control. Maybe revise to “step-by-step foot placement control was not tightened”.

Introduction:
Very well-written. No changes suggested.

Methods:

Line 141, what is “conform Hof”?

Line 217, I assume COMpos and COMvel are vectors consisting of x, y, and z components? Please elaborate on this.

Line 224, there is a superscript “|” over the word “seconds” and it is not clear what this means. This shows up in other places in the paper as well. Please clarify.

Line 231, contralateral foot is also at midstance?

Line 233, stance foot is also at midstance? In general, please make sure that if states are being measured at a particular gait event, that is mentioned clearly.

Line 233, not sure what the word “predictors” refers to.

Line 238, why is the EMG variable subscripted “swing”? Was the EMG only calculated at swing? The result figures with EMG indicate it was calculated throughout the stride cycle.

Please state If FP2 and the EMG variables in equation 2 are demeaned or not somewhere in the methods section.

In equation 1, if CoM_pos(i) and CoM_vel(i) are written with (i) to indicate step cycle i, should FP also not be FP(i) and not just FP? I have a similar question about equations 2: to my understanding, the EMG are also for one step cycle, so should that also not be (i) ?

Results:

Figure 3 caption states that medial burst is greater than lateral burst, is this difference significant? It looks very small from the figure.

In figures 3 and 4, the y axis does not have any number scale.

Figure 4 caption says “higher emg activity during early swing”, but this appears to be true only for slow speed. Please clarify this in the caption itself.

Line 335, please state the R^2 value there itself. Did Rankin et al. have a similarly low R^2? Please discuss what this could mean.

Discussion:
The discussion is generally very well written and the interpretations are made carefully.

In line 395, maybe use the word “relaxed” instead of “adapted”, to contrast with “tightened"?

Line 414, unclear what “serially coordinated as a balance response” means, please clarify

Line 420, could you elaborate on why you say “this does not necessarily imply more accurate control”

Conclusion:

In line 511, the use of the word “other” seems to indicate that foot placement control compensation was clearly eliminated in this study. However, as the author admits, this is not the case. It is possible that the LeSchuh did not allow for easy foot placement modulation. This can be explored with further experiments to see if the foot placement control compensates with practice with LeSchuh. Please add this caveat in the conclusion.
